# Acute presentation of vasospastic angina induced by oral capecitabine: a case report

**DOI:** 10.1186/1752-1947-8-18

**Published:** 2014-01-15

**Authors:** Christos Golias, Georgios Dimitriadis, Dimokritos Dimitriadis, Christos Graidis, Ilias Dimitrelos, Afroditi Tsiakou, Theodosis Bitsis, Konstantinos Charalabopoulos

**Affiliations:** 1 Department of Physiology, Clinical Unit, Medical Faculty, Democritus University of Thrace, Panepistimioupoli, Dragana 68100 Alexandroupolis, Greece; 2 Department of Cardiology, Serres State Hospital, 2nd km E.O Serrwn-Dramas, Serres 62100 Greece; 3 Department of Cardiology, Preveza State Hospital, Selefkias 2, Preveza 48100 Greece; 4 Department of Interventional Cardiology, Kyanous Stavros Hospital, Vizyis-Vyzantos 1, Thessaloniki 54636 Greece

**Keywords:** Acute coronary syndrome, Capecitabine, Cardiotoxicity, Vasospastic angina

## Abstract

**Introduction:**

Oral capecitabine is an oral prodrug of 5-fluorouracil that has been integrated into the management of multiple cancer types because of the convenience of administration and its efficacy compared with 5-fluorouracil. Capecitabine mimics the pharmacokinetics of intravenous 5-fluorouracil. While cardiac events associated with the use of 5-fluorouracil are a well-known side effect, capecitabine-induced cardiotoxicity has only been rarely reported.

**Case presentation:**

We present a case of a 46-year-old woman of Greek ethnicity who presented to our institution with an operated gastric sarcoma who experienced capecitabine-induced vasospastic angina. Primarily a clinical diagnosis of a possible acute coronary syndrome was proposed and the patient was admitted to the hospital for further investigation which was proved between normal limits. After a witnessed episode of angina, her prior history of capecitabine intake and an undertaken further imaging investigation we associated anginal symptoms and signs with vasospastic angina induced by capecitabine 36 hours prior to hospital admission.

**Conclusion:**

Cardiologists should be aware of the potential cardiac hazards of capecitabine, especially in patients with cardiovascular risk factors. Due to the increasing usage of capecitabine during the last years, patients should be warned for the possibility of chest pain, particularly during the first few days of capecitabine treatment. Specifically, patients developing acute coronary syndrome should not be retreated with capecitabine. On the other hand, due to its promising antitumoral efficacy, its use should not be discouraged.

## Introduction

Oral capecitabine is an oral prodrug of 5-fluorouracil (5-FU) that has been integrated into the management of multiple cancer types. Capecitabine, a thymidine phosphorylase– activated fluoropyrimidine carbamate, is absorbed by the gastrointestinal tract and metabolized to 5-FU by a cascade of three enzymes. It is currently considered the only universally approved orally administered 5-FU prodrug. It belongs to a new generation of orally administered fluoropyrimidines. It has been developed because of the clinical need for efficient, tolerable and convenient agents that do not require continuous infusion. Capecitabine is not a cytotoxic drug itself; by a three-step enzymatic cascade, it is converted to 5-FU mainly within human cancer cells. Capecitabine is converted by carboxylesterase in the liver to 5′-deoxy-5-fluorocytidine and by cytidine deaminase in the liver and tumor tissue to 5′-deoxy-5-fluorouridine. Furthermore, it is converted by thymidine phosphorylase to 5-FU in tumor tissue. Thymidine phosphorylase is found in higher concentrations in tumor tissue than in normal tissue and is upregulated by radiation in tumor tissue, but not in normal tissue. Thus, oral capecitabine can result in higher intratumoral and lower systemic 5-FU concentration than bolus delivery of 5-FU. The drug compares favorably with 5-FU in patients with colorectal cancer and breast cancer, and it has a better toxicity profile with mainly gastrointestinal and dermatologic effects. Capecitabine shows antineoplastic activity and synergy with other cytotoxic agents, including cyclophosphamide and docetaxel, in animal models. Bioavailability after oral administration is close to 100%. Although patients may receive the drug orally in the convenience of their own homes, the key to successful management of capecitabine is the clinician’s awareness of its severe, but low in incidence, adverse effects and the patient’s education, emphasizing compliance with the treatment plan, adverse effect prevention and timely recognition of the drug’s toxicities. This improved therapeutic index, along with more favorable pharmacokinetics (similar to those of protracted infusion of 5-FU) and convenient oral administration without the need for central venous access and an ambulatory infusion pump, make capecitabine particularly appealing to use.

In particular, although cardiac events associated with the use of 5-FU are a well- known side effect, capecitabine-induced cardiotoxicity has only rarely been reported [1-4]. Walko *et al*. compiled a review of reports in the literature describing adverse effects observed in capecitabine-treated patients [5]. Chest pain occurred in 6% of 758 breast and colorectal cancer patients treated with capecitabine at a dose of 2500mg/m^2^ per day in two divided doses for 14 days followed by 1 week of rest. The authors concluded that this complication is more frequent in patients who have a history of coronary artery disease and recommended close monitoring for cardiac abnormalities during therapy. Herein we present the case of a 46-year-old woman with an operated gastric sarcoma who presented to our institution with capecitabine-induced vasospastic angina.

## Case presentation

**Figure 1:**
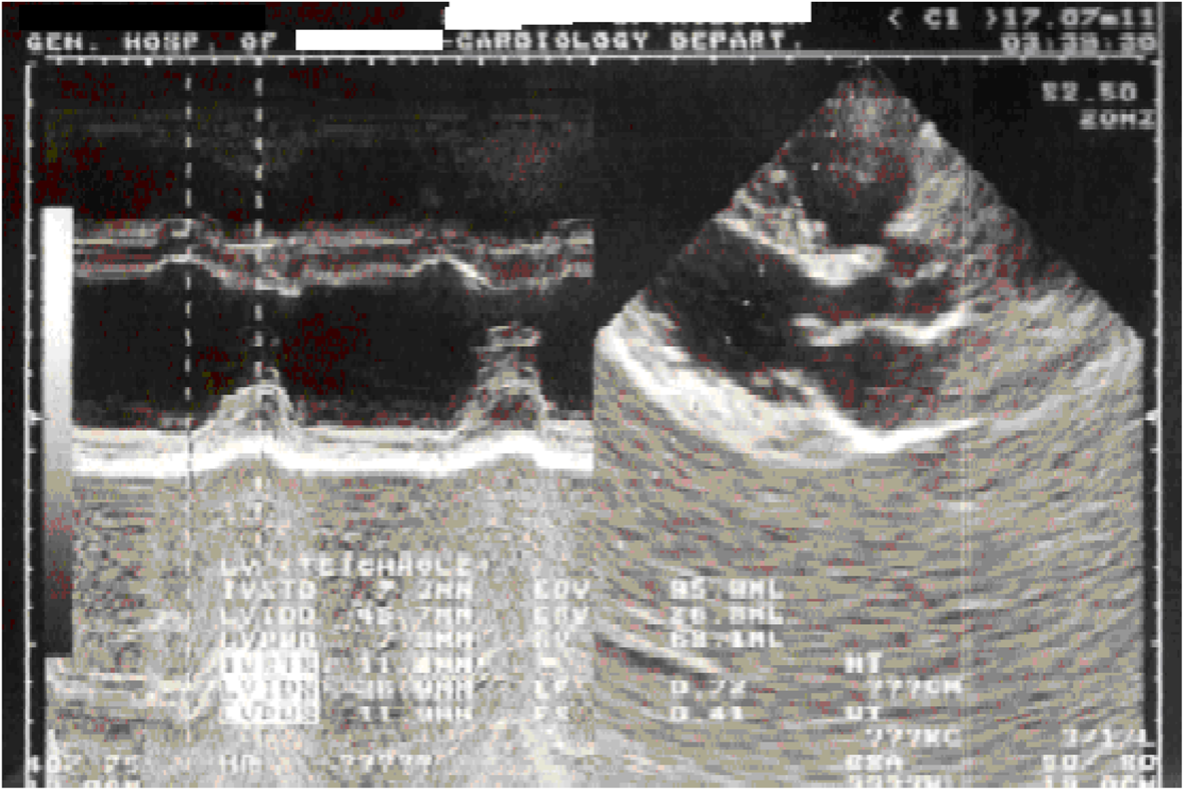
Echocardiogram obtained at the time of admission.

**Figure 2:**
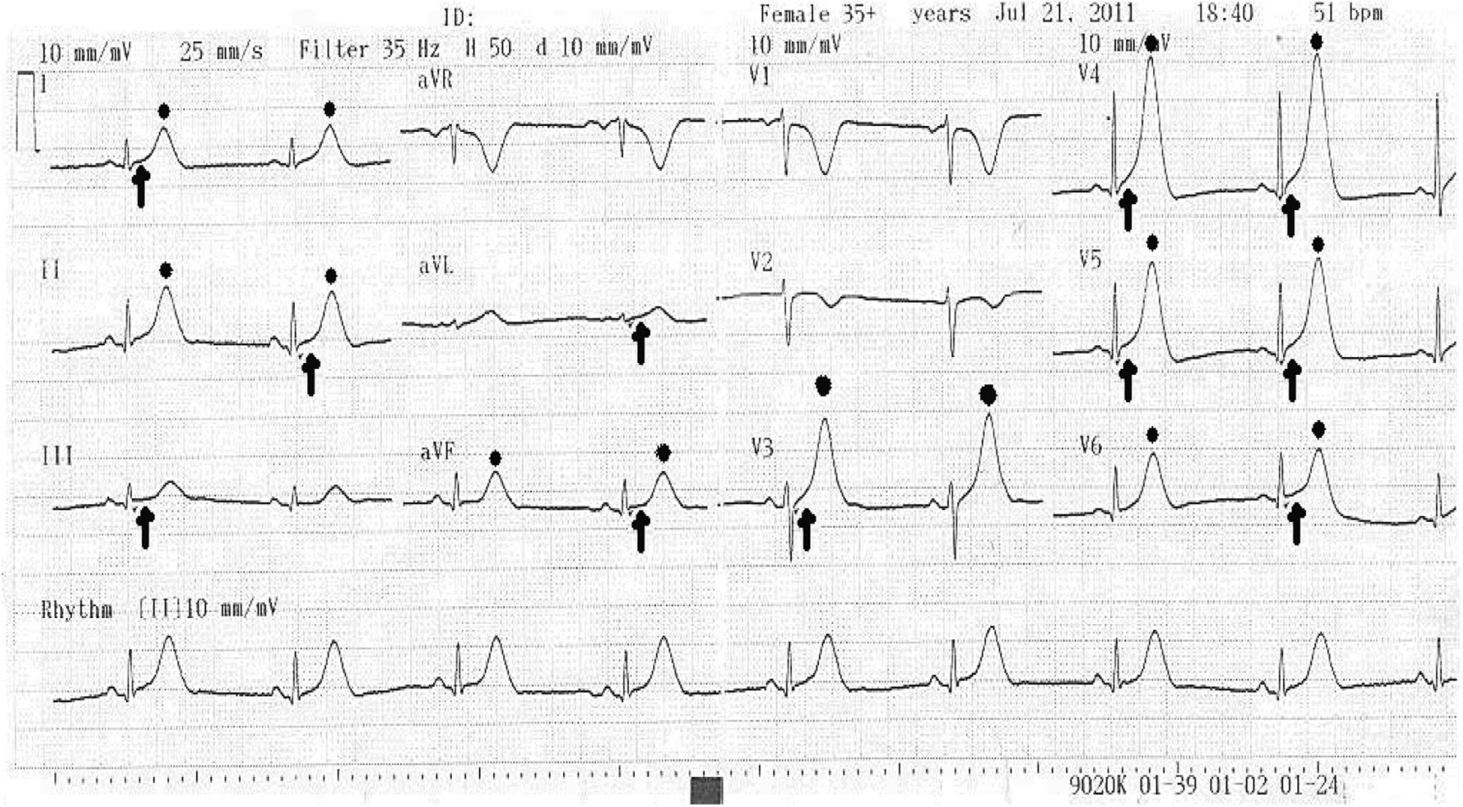
**Electrocardiogram taken during the anginal episode in the coronary intensive care unit.** Sinus bradycardia (50 beats per minute (bpm)) with diffuse ST-segment elevation (arrows) in the anterior leads (V3 to V6) and inferolateral leads (I, II, III, augmented vector left and augmented vector foot) and peaked T-waves (dots) at the same leads are suggestive of transmural ischemia.

**Figure 3:**
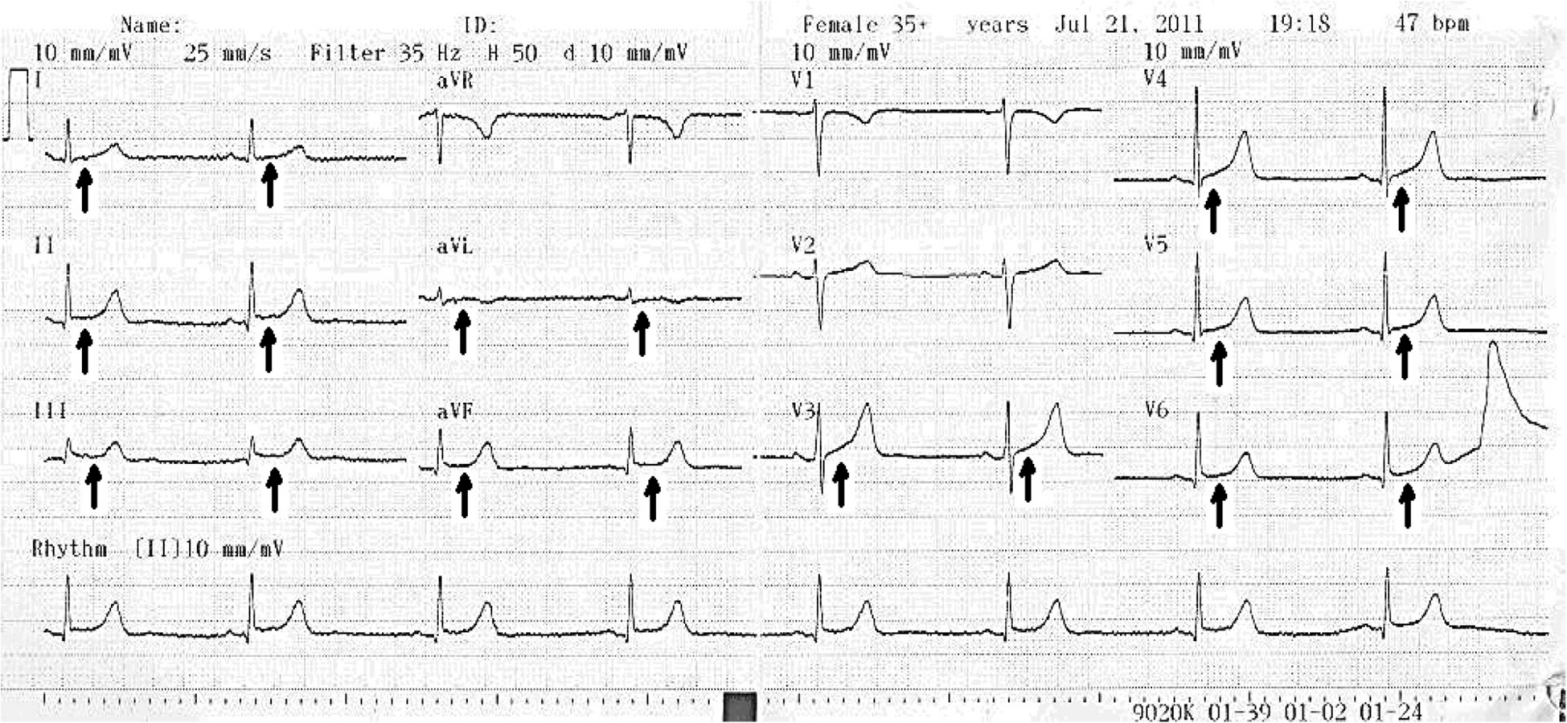
**Electrocardiogram taken 20 minutes after the patient’s pain resolved.** Arrows show progressive recovery of ventricular repolarization abnormalities.

**Figure 4:**
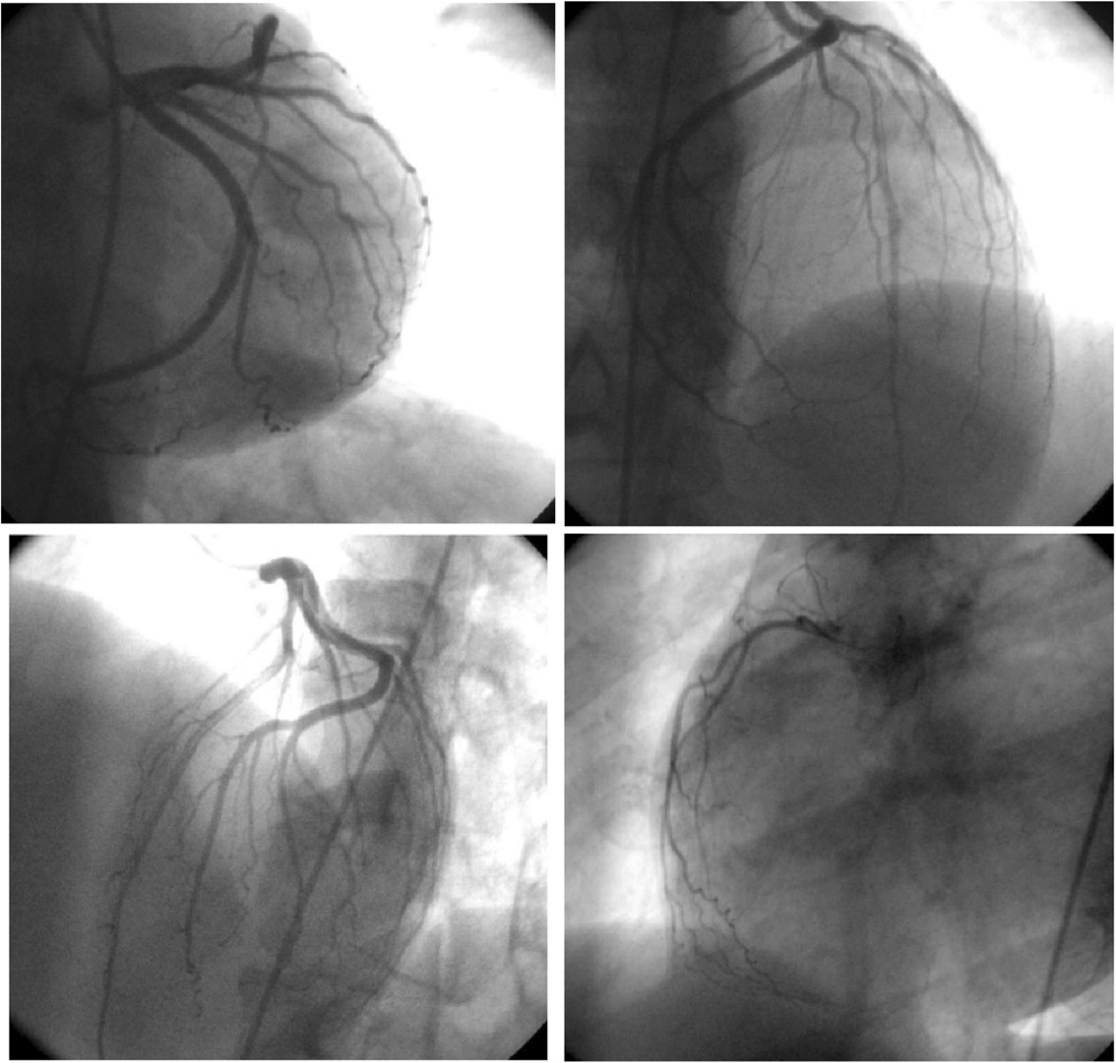
Coronary angiography images showing normal epicardial coronary artery tree and normal left ventricular function.

A 46-year-old woman of Greek ethnicity was admitted to the emergency room at our institution because of a retrosternal pain episode 90 minutes before admission that lasted approximately 10 minutes. Additionally, the patient reported two transient episodes of chest pain during the previous 24 hours. The duration of each episode was estimated to be between two and ten minutes, and spontaneous resolution was reported. During these episodes, the symptoms included chest discomfort with radiation to the back, malaise, nausea and sweating. Our patient had no prior history of cardiac disease, coagulation disorders or drug abuse, and she mentioned only one cardiovascular risk factor (smoking). From her medical history, we found that she is a heterozygous carrier of β-thalassemia. She reported having gastric sarcoma with metastatic peritoneal infiltrates. We had performed a subtotal gastrectomy on her for this reason 2 months prior to her current presentation. Treatment with oral capecitabine (1500mg twice daily) was initiated 48 hours before she was admitted to our hospital. The echocardiogram taken in the emergency room was within the normal limits, including a sinus heart rate of about 62 beats per minute (bpm) and physiological hemodynamic parameters and negative biochemical markers for myocardial necrosis (including cardiac troponin I (CTnI), creatine phosphokinase, creatine kinase isoenzyme MB, lactate dehydrogenase, and serum glutamic oxaloacetic transaminase). She was transferred to the coronary intensive care unit (ICU) for further observation. The echocardiogram, which was performed when the patient was pain-free, revealed normal ventricular volume, no segmentary wall hypokinesia with a left ventricular ejection fraction of 72%, normal diastolic heart function, normal valve flow and absence of pericardial effusion (Figure 1). Taking into account the patient’s Thrombolysis in Myocardial Infarction score for unstable angina, which was very low, she continued taking capecitabine under uninterrupted monitoring [6]. The cardiac enzymes for the next 24 hours were also negative for myocardial necrosis, and consecutive electrocardiogram (ECG) readings showed no repolarization abnormalities. Thirty-six hours after the last episode, while on telemetry, the patient had a witnessed episode of heavy retrosternal chest discomfort. A new ECG was taken immediately and revealed sinus bradycardia (50bpm) with diffuse ST-segment elevation in the anterior leads (V3 to V6) and inferolateral leads (I, II, III, augmented vector left (aVL) and augmented vector foot (aVF)) and peaked T-waves at the same leads (Figure 2), suggestive of transmural ischemia. Her blood pressure was 150/90mmHg. Her pain subsided after continuous infusion of nitroglycerin (4mg/h) was initiated and a 5mg capsule of nifedipine was given to the patient sublingually. The patient was symptomless within 10 minutes. The ECG that was taken 40 minutes afterward showed progressive recovery of ventricular repolarization abnormalities (Figure 3). Daily ECGs confirmed a normal repolarization pattern. Serum cardiac markers, including CTnI, remained within the normal range. Coronary and ventricular angiography performed 48 hours later revealed a normal epicardial coronary artery tree and normal left ventricular function (Figure 4). She was discharged without active coronary therapy. To control the oncological disease, intravenous cisplatin treatment was initiated.

## Discussion

Capecitabine is an oral prodrug that is converted through a three-stage enzymatic reaction to 5-FU primarily in liver and tumor cells. Through the years, it has gained popularity as well as support from the oncologic community because of its efficacy, ease of administration and milder toxicity profile. The cardiotoxic effects of intravenously administered 5-FU pyrimidine analogues have been well-described in the literature, with a reported incidence ranging from 1% to 68% [7,8], whereas the cardiotoxic effects of capecitabine, the oral formulation of 5-FU, are less familiar to cardiologists. These manifestations include acute coronary syndrome (ACS), heart failure, hypertension, hypotension, cardiomyopathy and arrhythmias. Myocardial injury, thrombogenic effects, immunoallergic reaction and coronary vasospasm have all been implicated in the mechanism underlying 5-FU cardiotoxicity [9,10]. Capecitabine toxicity is thought to have the same etiology as the toxicity of 5-FU, although capecitabine and its metabolites are minimally cytotoxic *in vitro* compared with 5-FU [11]. Our present case report demonstrates that capecitabine can induce ACS. Although the true incidence of this complication still has not been defined, chest pain during capecitabine therapy has been reported in 6% of patients [12]. Acute coronary thrombosis cannot completely be excluded by angiography. Coronary vasospasm is another mechanism that might possibly be involved. However, the efficacy of vasodilating drugs given prophylactically to patients with previous episodes of chest pain during 5-FU treatment has been reported, but with inconsistent results [13].

## Conclusion

Capecitabine should be considered a drug with cardiotoxic potential [14], even in the absence of prior cardiac history. It can induce coronary spasm at the macro- or microvascular level [15]. Cardiologists should be aware of its potential cardiac hazards, which might be manifested by coronary angiospasm, especially in patients with cardiovascular risk factors. Because of the increasing use of capecitabine during the past several years, patients should be warned and informed about the possibility of chest pain and other possible emergent anginal symptoms, particularly during the first few days of capecitabine treatment. Most importantly, patients who develop ACS should not be retreated with capecitabine. However, because of capecitabine’s promising antitumoral efficacy, its use should not be discouraged.

## Consent

Written informed consent was obtained from the patient for publication of this case report and any accompanying images. A copy of the written consent is available for review by the Editor-in-Chief of this journal.

## Competing interests

The authors declare that they have no competing interests.

## Authors’ contributions

CGo made substantial contributions to the conception and design of this report, drafting the manuscript and revising it critically. He gave final approval of the version to be published. GD made substantial contributions to the conception of this report, drafting the manuscript and revising it critically and analyzing and interpreting echocardiographic data. DD and CGr made substantial contributions to drafting the manuscript and performing analysis and interpretation of coronary angiography data. AT examined the patient and analyzed and interpreted the data regarding hospital admission. TB and ID made substantial contributions to the collection and acquisition of data and drafting the manuscript. KC made substantial contribution to the conception and design of this report; acquisition, analysis and interpretation of data. He was involved in revising the manuscript critically and gave final approval of the version to be published. All authors read and approved the final manuscript.
